# Vehicle Density Based Forwarding Protocol for Safety Message Broadcast in VANET

**DOI:** 10.1155/2014/584164

**Published:** 2014-07-10

**Authors:** Jiawei Huang, Yi Huang, Jianxin Wang

**Affiliations:** School of Information Science and Engineering, Central South University, Changsha 410083, China

## Abstract

In vehicular ad hoc networks (VANETs), the medium access control (MAC) protocol is of great importance to provide time-critical safety applications. Contemporary multihop broadcast protocols in VANETs usually choose the farthest node in broadcast range as the forwarder to reduce the number of forwarding hops. However, in this paper, we demonstrate that the farthest forwarder may experience large contention delay in case of high vehicle density. We propose an IEEE 802.11-based multihop broadcast protocol VDF to address the issue of emergency message dissemination. To achieve the tradeoff between contention delay and forwarding hops, VDF adaptably chooses the forwarder according to the vehicle density. Simulation results show that, due to its ability to decrease the transmission collisions, the proposed protocol can provide significantly lower broadcast delay.

## 1. Introduction

During the last decade, the intelligent transportation systems (ITSs) have used advanced wireless communication technologies to enhance current road transportation systems. Most of ITS applications utilize vehicular ad hoc networks (VANETs) to provide the communications between vehicle-to-infrastructure (V2I) and vehicle-to-vehicle (V2V) [1]. As a kind of self-organizing wireless networks, VANET chooses the suitable candidate vehicle as the forwarder to accomplish the multihop data delivery.

The accident detection and avoidance by disseminating safety messages are considered as one of the most important services of VANETs. When accident happens, the safety message should be broadcasted through the following vehicles (namely, a platoon) covering a specific area of few kilometers [2–4]. Lowering the dissemination delay between the time of an accident event and the time at which all vehicles of the following platoon receive the emergency message, the probability of chain collisions can be reduced. Since the wireless transmission range is limited (about 250 m), the vehicles have to relay the safety messages by multihop broadcasting. In the case of accident, there exist two issues. The first is how to accomplish the multihop broadcasting in very tight message delivery time, typically few hundreds of milliseconds [5]. The second one is that the messages should be delivered to all vehicles with very high delivery reliability.

Due to the high mobility and restricted mobility patterns of vehicles, it is challenging work to design emergency message dissemination scheme with low delay and high reliability. Because the wireless channel is shared by all vehicles, the flooding broadcasting will lead to transmission contention and collision among neighboring vehicles, which degrades reliability and efficiency [6–8]. Many researchers have widely investigated the problem of disseminating safety messages in IEEE 802.11p protocol, which is MAC-layer standard in VANET [9]. Most of the proposed multihop broadcast protocols use the geographical information to avoid the broadcast storm problem. In these protocols, each vehicle is aware of its own position by the GPS devices. To reduce the number of forwarding hops in multihop broadcast, the farthest node in broadcast range is chosen as the forwarder. Consequently, the end-to-end broadcast delay is decreased.

In this paper, we use mathematical analysis and simulation results to demonstrate that, when the vehicle density is large, the farthest forwarder may experience large number of collisions, which bring about the high contention delay. Thus, we propose an efficient vehicle density based forwarding (VDF) protocol for safety message dissemination in multihop VANETs. The protocol adaptably chooses the forwarder according to the vehicle density to obtain the good tradeoff between contention delay and forwarding hops.

The remainder of this paper is organized as follows. In Section 2, we present a brief overview of the related broadcast protocols. To investigate the problem of current protocols in detail, the mathematical analysis and simulation results are given in Section 3. The proposed protocol is described in Section 4.  Section 5 compares and analyzes the protocol performances by the NS2 simulations. Finally, we draw conclusions in Section 6.

## 2. Related Work

In wireless networks, how to achieve high efficient multihop broadcast or coverage is a challenging task [10, 11]. In the multihop broadcasting, when a source vehicle broadcasts safety message, some of the vehicles within the vicinity of the source will become the next forwarding vehicles and perform relaying by rebroadcasting the message further. In the naive pure flooding scheme [12], each vehicle rebroadcasts the packet. It is obvious that, when the network becomes denser, the same message will be rebroadcasted more redundantly. The limited wireless channel bandwidth is wasted. Moreover, the packet collision problem becomes severe since a large number of vehicles in the same vicinity may rebroadcast the message at the same time. To solve the broad storm problem, the common method is adjusting the broadcast delay or probability. In this way, the channel contention brought by the flooding broadcast is mitigated.

Generally, in the delay-based broadcasting protocols, the farthest candidate forwarding vehicle is given the shortest broadcast delay. An efficient 802.11-based protocol called urban multihop broadcast (UMB) is proposed in [13]. UMB assigns each node with the specified broadcasting delay, which is determined by the distance between the vehicle and the transmitter. The lower broadcasting delay is assigned to the vehicle that is farther away from the transmitter. Therefore, the vehicle with the lowest delay has the highest priority to rebroadcast the message. At the same, when receiving the rebroadcasted message, the other vehicles cancel the retransmission process.

As another typical delay-based protocol, ReC [14] also uses geographical information to select the forwarding vehicles. In ReC, the selected forwarder is the nearest vehicle to the centroid of neighboring vehicles that have not received the message. Once receiving the message, the selected forwarder retransmits immediately. Thus, the unnecessary retransmission is reduced, while at the same time the forwarder can retransmit the message without delay. However, due to the vehicle's high mobility, there exists a great practical difficulty that ReC requires a complete and continuously updated knowledge of the neighboring vehicles.

To avoid the inaccurate transmission range estimation in highly mobile environment, JIVCA [15] utilizes the hello messages to get the real-time position information of other vehicles around. With the neighbor position information, JIVCA continuously updates its transmission range estimation. Similar to the other delay-based protocols, JIVCA prioritizes farther vehicles in forwarding messages. In particular, the broadcast delay is computed based on the contention window (CW) in IEEE 802.11p MAC protocols. The farther vehicle has smaller CW and thus will have lower waiting delay before relaying the message.

In probabilistic-based broadcasting, a different rebroadcast probability is assigned to each receiving vehicle [16–18]. In [12], the weighted *p*-persistence protocol is proposed. The vehicle that receives message computes its own rebroadcast probability based on the distance between itself and the transmitter. The rebroadcast probability becomes larger as the distance between the vehicle and the transmitter increases. The farthest node from the sender has the highest chance of rebroadcasting the message firstly. Since only some vehicles will rebroadcast the message, the number of redundant messages and channel collisions is decreased.

To maximize the forwarding speed of safety messages, both delay-based and probabilistic-based broadcasting protocols give the highest priority to farthest vehicle to relay message. Though the number of forwarding hop between the sender and the last receiver is reduced, there exists the contention problem between the forwarder vehicle and the vehicles outside of the transmission range. Since the farthest vehicle is selected as the forwarder, when the forwarder rebroadcasts the message, large number of vehicles may contend with the forwarder, which enlarges the backoff delay for the usage of wireless channel. Furthermore, when the vehicle density increases, the contention delay will become larger and the end-to-end multihop broadcast performance is degraded.

## 3. Problem Analysis

In this section, we first describe the system assumption. Then, the mathematical model and analysis simulation are used to demonstrate contention problem when the farthest vehicle is selected as the forwarder.

**Figure 1:**
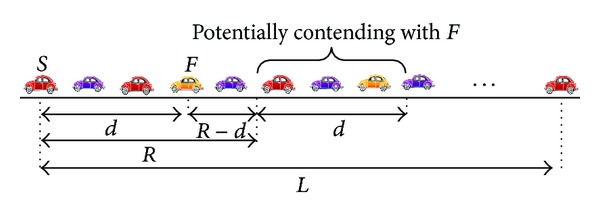
Network scenario.

The considered network scenario is in a multilane highway environment as shown in Figure 1. Since the transmission range *R* is much larger than the road width, the network scenario can be simplified as a one-dimensional VANET with road length of *L*. The vehicles are uniformly distributed on the road and the vehicle density is *α*. In each relay, the distance between the sender *S* and forwarder *F* is hop distance *d*. We assume all of the vehicles are equipped with GPS to acquire their own positions.

Here, we analyze the end-to-end broadcast delay *T* considering the transmission range *R*, hop distance *d*, and vehicle density *α*. We characterize transmission states of the forwarder *F*. As shown in Figure 1, since *F* is selected as the forwarder, *F* will rebroadcast the message first. Hence, we assume that there is no other contending vehicle in the transmission range *R* of sender *S*. However, the vehicles outside of the transmission range are potentially contending with *F*. The number of potential contending vehicles is calculated as *αd*.

We model the backoff procedure of the IEEE 802.11p as a *p*-persistent CSMA/CA. Different from the binary exponential backoff in IEEE 802.11p, the backoff interval of the *p*-persistent CSMA/CA is sampled from a geometric distribution with transmission probability *p*. The *p*-persistent CSMA/CA provides a very close approximation to the IEEE 802.11 protocol [19, 20], and the memoryless backoff algorithm makes it suitable for mathematical analysis.

**Figure 2:**
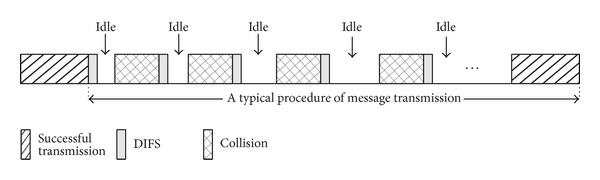
A typical procedure of message transmission.

We observe the procedure before every successful message transmission.  Figure 2 shows that collisions and idle periods may occur before a successful transmission. The collision's reason is that more than one vehicle transmits at the same time slot. The idle period is a time interval (expressed in number of time slots *σ*) in which the transmission medium remains free of any transmission due to the backoff algorithm.

Assuming that *F* experiences *n* collisions before a successful transmission, therefore, we have the probability of successful transmission *P*_*s*_ and collision probability *P*_*c*_ as

(1) $$\begin{array}{c} P_{s}=\frac{\alpha dp{\left(1-p\right)}^{\alpha d-1}}{1-{\left(1-p\right)}^{\alpha d}},\\ P_{c}=\frac{1-{\left(1-p\right)}^{\alpha d}-\alpha dp{\left(1-p\right)}^{\alpha d-1}}{1-{\left(1-p\right)}^{\alpha d}}, \end{array}$$

where the transmission probability *p* can be calculated with the minimum value CW_min⁡_ of contention window as

(2) $$\begin{array}{c} p=\frac{2}{\text{C}{\text{W}}_{\min}+1}. \end{array}$$

From (1), the expected value *E*[*N*_*c*_] of collision number *n* before a successful transmission can be obtained as

(3) $$\begin{array}{c} E\left[N_{c}\right]=\frac{P_{c}}{P_{s}}=\frac{1-{\left(1-p\right)}^{\alpha d}}{\alpha dp{\left(1-p\right)}^{\alpha d-1}}-1. \end{array}$$

For each transmission collision, the collision time *T*_*c*_ includes the message's transmission time *σm* and the DIFS time *σD*. Therefore, the total collision time *T*_col_ before a successful transmission is

(4) $$\begin{array}{c} T_{\text{col}}=E\left[N_{c}\right]T_{c}=\sigma \left[\frac{1-{\left(1-p\right)}^{\alpha d}}{\alpha dp{\left(1-p\right)}^{\alpha d-1}}-1\right]\left(m+D\right). \end{array}$$

As shown in Figure 2, since a collision is just between two idle periods, the expected number *E*[*N*_*i*_] of idle period is

(5) $$\begin{array}{c} E\left[N_{i}\right]=E\left[N_{c}\right]+1=\frac{1-{\left(1-p\right)}^{\alpha d}}{\alpha dp{\left(1-p\right)}^{\alpha d-1}}. \end{array}$$

The number of time slots *T*_*i*_ in each idle period is determined by the transmission probability *p* and contending vehicle number *αd*. The expected value of *E*[*T*_*i*_] is

(6) $$\begin{array}{c} E\left[T_{i}\right]=\sigma \left[1-{\left(1-p\right)}^{\alpha d}\right]\overset{\infty }{\underset{i=0}{\sum }}i{\left(1-p\right)}^{\alpha di}=\frac{\sigma {\left(1-p\right)}^{\alpha d}}{1-{\left(1-p\right)}^{\alpha d}}. \end{array}$$

Thus, the total collision time *T*_idle_ before a successful transmission can be calculated as

(7) $$\begin{array}{c} T_{\text{idle}}=E\left[N_{i}\right]E\left[T_{i}\right]=\frac{\sigma \left(1-p\right)}{\alpha dp}. \end{array}$$

The successful transmission time *T*_trans⁡_ is

(8) $$\begin{array}{c} T_{\mathrm{trans}}=\sigma \left(m+D\right). \end{array}$$

Since the one-hop transmission time *T*_hop_ is composed of the collision time *T*_col_, idle time *T*_idle_, and successful transmission time *T*_trans⁡_, we have

(9) Thop=Tcol+Tidle+Ttrans⁡=σ[m+D−(m+D−1)(1−p)αd]αdp(1−p)αd−1.

From the source vehicle of message to the last receiver, the end-to-end multihop broadcast is made up of *L*/*d* relay hops. The end-to-end broadcast delay *T* can be expressed as

(10) $$\begin{array}{c} T=\frac{\sigma L\left[m+D-\left(m+D-1\right){\left(1-p\right)}^{\alpha d}\right]}{\alpha d^{2}p{\left(1-p\right)}^{\alpha d-1}}. \end{array}$$

We validate our analysis using MATLAB and NS2 [21] simulations. We simulate a VANET scenario as shown in Figure 1, in which all vehicles are using IEEE 802.11p as the MAC-layer protocol. The whole 5 km road segment is composed of four lanes. Along each lane vehicles are uniformly deployed and moving at the constant velocity. The message size *m* is 1,000 Bytes, which corresponds to the transmission time of 32 time slots giving the 1 Mbps wireless transmission rate.  Table 1 shows the road traffic parameters and MAC protocol settings, which are used for both the simulations and the analysis. It should be noted that the vehicle densities are set as 25, 100, and 175 vel/km/lane for different scenarios. The corresponding values of vehicle density *α* are 0.1, 0.4, and 0.7 vel/m, respectively.

**Figure 3:**
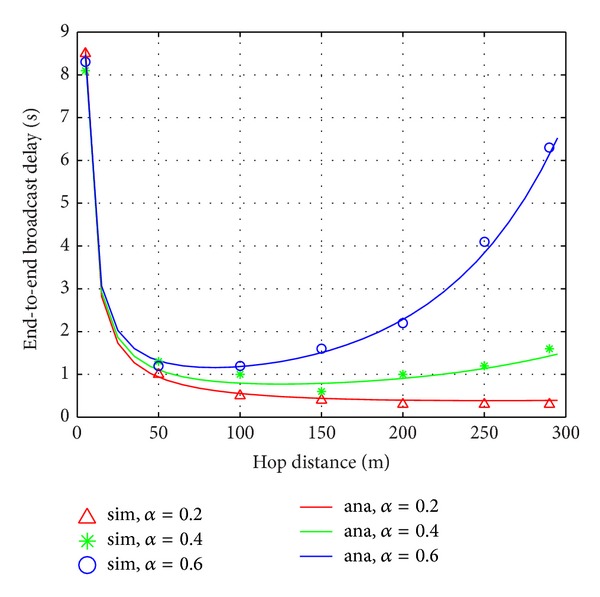
End-to-end broadcast delay with different hop distance.

In Figure 3, we change the hop distance *d* from 5 m to 290 m. The larger hop distance, the farther vehicle is chosen as forwarder. From both mathematical and simulation results, we observe that the end-to-end broadcast delay *T* is sensitive to the vehicle density *α*. When the vehicle density is small (*α* = 0.1 vel/m), the end-to-end broadcast delay always decreases if the hop distance becomes larger. The reason is that when the contention is low, the message dissemination will be accelerated if the farthest vehicle is the forwarder. However, when the vehicle density is large, the channel contention for the forwarder becomes heavy if the forwarder is still the farthest vehicle. This, as a consequence, results in high message collision ratio and long contention delay. Figure 3 shows that, when *α* is 0.7 vel/m, the end-to-end broadcast delay becomes very high if the hop distance is large.

Based on this observation, the optimal hop distance between the sender *S* and forwarder *F*, *d*_opt_, which minimizes the value of end-to-end broadcast delay *T*, can be obtained by equating the first derivative of *T* with respect to *d* to zero. As presented in the following section, in our proposed protocol, the *d*_opt_ value is used to tune the contention window size to reach the desired performance.

## 4. Vehicle Density Based Forwarding

The basic ideal of our proposed VDF protocol is to select the forwarder *F* with the optimal hop distance *d*_opt_ according to the vehicle density *α*. In the following, we present the design detail of VDF protocol with the pseudocode shown as Pseudocode 1.

In order to sense the vehicle density *α* in the transmission range *R*, each vehicle utilizes the beacon message to inform its neighboring vehicles. The information in beacon message includes the vehicle's identity and its position. When receiving the beacon message, the vehicle counts the vehicle number in its transmission range and then calculates the vehicle density *α*. Moreover, the vehicle can calculate the distance *d*_*f*_ from the current forwarder to itself with the help of GPS devices. As analyzed in the previous section, the given values of *σ*, *L*, *m*, *D*, *p*, and *α*, *d*_opt_ can be numerically computed. It should be noted that the maximum value of *d*_opt_ is the transmission range *R*.

By assigning different waiting times from the reception to rebroadcasting of the message, VDF prioritizes the best relaying vehicle with hop distance *d*_opt_ in forwarding the message. The waiting time is determined by the contention window in IEEE 802.11p MAC protocols.

Upon receiving the new safety message from the forwarder, each vehicle computes its own contention window CW as

(11) $$\begin{array}{c} \text{CW}=\left|\frac{d_{f}-d_{\text{opt}}}{R}\right|\times \left(\text{C}{\text{W}}_{\max}-\text{C}{\text{W}}_{\min}\right)+\text{C}{\text{W}}_{\min}, \end{array}$$

where CW_min⁡_ and CW_max⁡_ are the minimum and maximum contention window, respectively. From (11), it is clear that the vehicle with smaller value of |*d*_*f*_ − *d*_opt_| will have a smaller CW. That means that the vehicle will have shorter waiting time to transmit the message and, implicitly, higher probability to be selected as the forwarder.

During the waiting, the vehicle may receive the same message again. Then the vehicle stops trying to forward the message, because another vehicle with shorter waiting time already did it. If the waiting time expires without having received the same message from any other vehicle, the vehicle becomes the forwarder and rebroadcasts the message.

## 5. Performance Evaluation

In this section, we compare the performances of VDF with ReC and 802.11p by using NS2 simulator. As mentioned before, 802.11p protocol employs random backoff scheme without any distance prioritization. In the following, we give the performance metrics, simulation setup, and performance analysis.

### 5.1. Performance Metrics

#### 5.1.1. Broadcast Delay

The main focus of our proposed protocol lies in reducing broadcast delay, which is a crucial factor in time-critical safety applications. The first performance metric is message broadcast delay, which is defined as the dissemination delay of the safety message from the source vehicle to the last receiver. The faster the safety message propagates, the more efficient the corresponding protocol is in terms of satisfying the urgent delay requirement of emergency application.

#### 5.1.2. Broadcast Count

The message dissemination progress can be measured by the broadcast count, which includes both the success and failure broadcast. The larger hop distance implies the high channel conflict probability and thus larger number of failure broadcast. However, the smaller hop distance means small coverage range and more relay hops. Only by obtaining good tradeoff between the coverage range and the rebroadcast probability could the fast message dissemination progress be achieved.

### 5.2. Simulation Setup

In the performance evaluation, we model a straight highway with 4 unidirectional lanes, where the vehicles are uniformly deployed. All vehicles send the beacon messages to periodically announce their ID and position with the generation rate of 10 beacon/s. The beacon size is set to 100 B. Moreover, in order to simulate the communication traffic such as web chat applications, we assume all the vehicles send 1.5 KB data packets to their neighbors with the rate of 10 packets/s. The other road traffic parameters and MAC protocol settings are the same as that in Table 1.

To compare the various schemes, we simulate two typical network applications including accident alert and online game. In the accident alert application, the messages are generated only in the case of the abnormal behavior of some vehicles. Specifically, the simulations of this application include single source (Section 5.3) and multiple sources configurations (Section 5.4). In the single source case, only the first vehicle is chosen as the emergency message source. On the other hand, in the multiple sources configuration, a different number of vehicles are randomly selected as the message sources. During simulation, the source vehicles broadcast the emergency message backward to all the following vehicles along the 5 km highway.

The online game is one of the most popular applications in VANET. In the online game application, each player periodically generates multihop transmissions to the other players. The wireless channel is shared by all players in the same application. Moreover, the generation rate is very important for the player experience. Thus, we vary the transmission interval to test the performances under different congestion state of wireless channel (Section 5.5). In the simulations, each test is repeated 10 times. The average value of test results is calculated with 95% confidence intervals.

### 5.3. Accident Alert: Impact of Vehicle Density

We start our evaluation focusing on the performances with varying vehicle density. In this test, the vehicle density is increased from 50 to 250 vehicles per km. Moreover, only the first vehicle is the emergency message source in this test.

**Figure 4:**
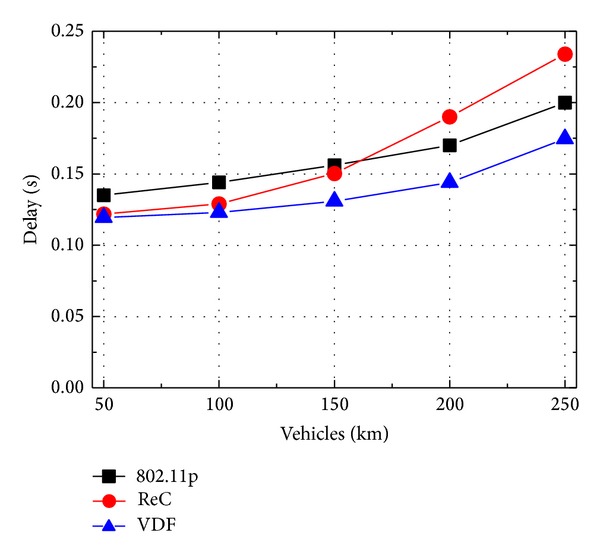
Accident alert: delay with different vehicle density.

Figure 4 shows the message broadcast delay against node density. Initially, when the vehicle density is only 50 vehicles per km, all three protocols obtain small delay. VDF experiences the delay close to ReC but 0.03 s smaller than 802.11p. The performance gap with 802.11p is the consequence of the fact that 802.11p randomly selects the forwarder and results in the larger average hop distance between the forwarders compared with ReC and VDF. As a result, the dissemination speed is reduced by 802.11p.

When the vehicle density increases, the message broadcast delay becomes higher with the larger conflict probability in wireless channel. However, the delay increasing of ReC is much faster than the other two protocols. When the vehicle density is 250 vehicles per km, the delay of ReC is about 34% and 15% higher than that of VDF and 802.11p, respectively. This result is explained by the fact that the larger the vehicle density, the higher the possibility of transmission collision as a single transmission range area hosts more vehicles. Though ReC selects the farthest vehicle as the forwarder to obtain the maximum coverage, it devotes more time to the collision resolution that incurs longer backoff time and thus large delay. Among the three protocols, VDF achieves the best delay performance as it adjusts the hop distance according to the vehicle density. When the vehicle density increases, VDF reduces the hop distance between the forwarders and alleviates the impact of high channel collision rate.

**Figure 5:**
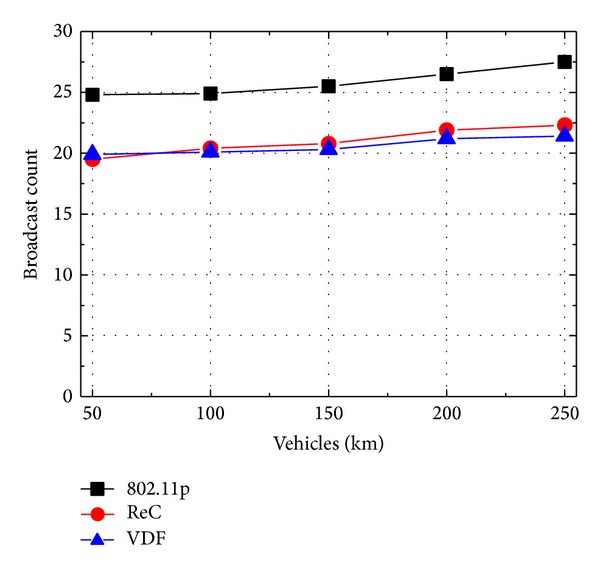
Accident alert: broadcast count with different vehicle density.

The results of broadcast count required to cover all vehicles are shown in Figure 5. 802.11p has the highest broadcast count as it randomly selects the forwarder vehicle and needs more relay hops to transmit the message to last vehicles. In contrast, with the target of providing the maximum coverage, the broadcast count of ReC is about 20% less than that of 802.11p. Compared with ReC, VDF obtains the nearly same performance though it does not always choose the farthest vehicle as the forwarder. Moreover, when the vehicle density is larger than 150 vehicles per km, VDF is even slightly lower than ReC. This is because that, when the channel contention becomes heavier, VDF reduces the coverage range and then gets the lower rebroadcast probability. Though the relay hops of VDF are more than ReC, VDF still obtains the smaller broadcast count because of the lower number of rebroadcasts.

### 5.4. Accident Alert: Impact of Source Number

In this evaluation, we test the protocol performances in the multiple sources configuration. 5% to 25% of randomly selected vehicles act as the message sources, which send messages backward to cover all vehicles. The vehicle density is fixed as 125 vehicles per km.

**Figure 6:**
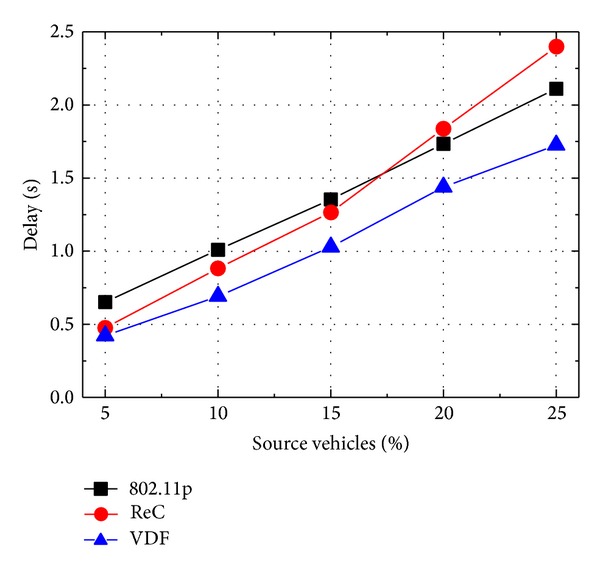
Accident alert: delay with different number of source vehicles.

In Figure 6, the message broadcast delay is shown for different number of message sources. For each protocol, the broadcast delay becomes larger with the increasing of number of message sources. This result is supported by the requirement of intensive collision resolution phases with increase in message sources. Moreover, under the heavy channel contention, it is seen that all the broadcast protocols exhibit much larger delay compared with the results of single source configuration. Because of the fixed hop distance in broadcast relay, ReC has the fastest increasing speed of message broadcast delay among the three protocols. Different from ReC, VDF selects the small coverage range to avoid the intensive channel contention. Therefore, VDF gets the best delay performance in three protocols.

**Figure 7:**
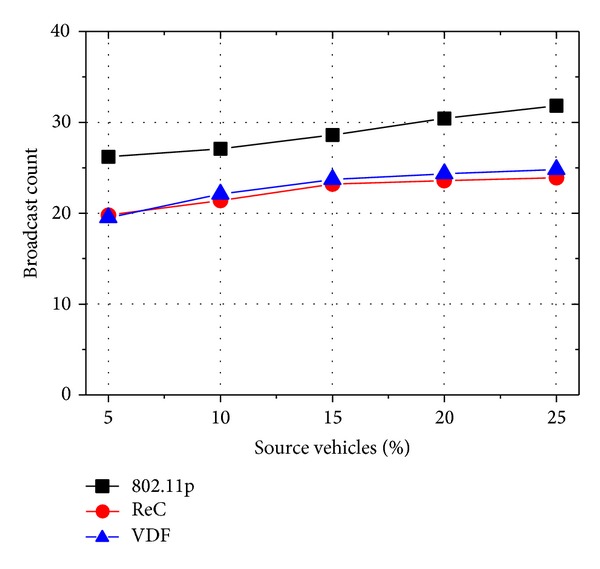
Accident alert: broadcast count with different number of source vehicles.

The comparison results for the number of broadcast count are shown in Figure 7. Once again, 802.11p suffers from the highest message broadcast count due to random manner in selecting broadcast forwarders. Both ReC and VDF obtain the lowest broadcast count by considering the coverage speed. It is noticed that, when the number of message sources becomes larger, since VDF experiences a smaller number of rebroadcast, VDF gets a slightly lower broadcast count than ReC.

### 5.5. Online Game

In the test of online game application, the vehicle density is set as 100 vehicles per km. We randomly select 50 vehicles as the players, which periodically generate 2000-Byte-sized packets to the other players. We evaluate the delay performances considering different generation intervals with each player. Specifically, the packets are generated at each vehicle every 10, 50, or 100 ms.

**Figure 8:**
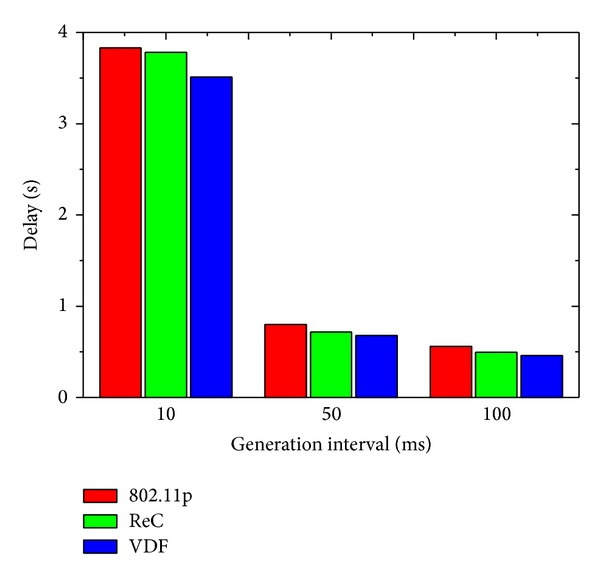
Online game: delay with different generation interval.

We measure the average value of game event delay, which is defined from the time that the player sends packet to the time that all the other players receive the packet.  Figure 8 presents the delay results with different generation intervals. It could be observed that, when the generation rate is 10 ms, the game event delay of all protocol is larger than 3.5 s. These results are attributed to the high packet generation rate and multitude of sources. If the generation interval is 50 ms or 100 ms, the game event delay drops to below 0.8 s. Compared with the case of 50 ms, the game event delay of 100 ms interval is only slightly lower. This indicates that the generation interval is not small enough to saturate the wireless channel. As expected, in the three protocols, VDF gets the lowest delay with all different generation intervals. This is a logic consequence of the fact that VDF decreases the coverage range to adapt to the high transmission interference.

## 6. Conclusion

We design and implement VDF, a vehicle density based forwarding protocol for safety message broadcast in VANET. By adaptively selecting the forwarder node according to the vehicle density, VDF alleviates the heavy wireless channel contention. Thus, VDF achieves the low broadcast delay and small broadcast count in multihop broadcast. By using NS2 simulations, we show that VDF has better performance than the existing message broadcast protocols in two typical network applications including accident alert and online game.

In the future, in order to avoid the impact of highly dynamic circumstances, we will design the backoff algorithm based on motion prediction of vehicle nodes. Moreover, we will test the protocol performance with large scale of testbed experiment.

**Pseudocode 1:**
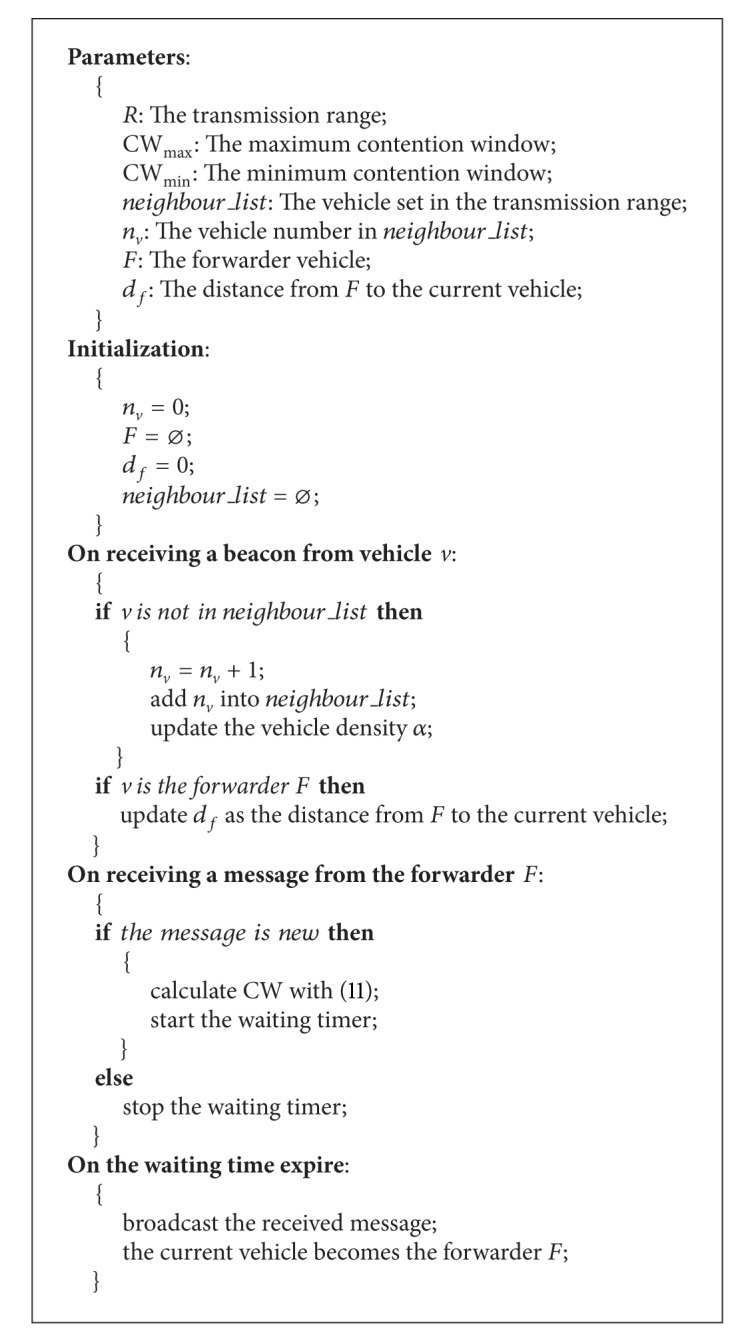
Pseudocode of VDF.

## Figures and Tables

**Table 1:** Road traffic parameters and MAC protocol setting.

Parameter and setting	Value
Vehicle density α	25, 100, and 175 vel/km/lane
Vehicle speed *v*	80 km/h
Road length *L*	5000 m
Number of lanes	4
Transmission range *R*	300 m
Channel propagation	Two-ray ground
Time slot σ	20 μ s
DIFS time σ*D*	50 μ s
CW_min⁡_	31 slots
CW_max⁡_	1023 slots
Wireless transmission rate *r*	1 Mbps
Simulation time	70 sec
